# Sex Differences in the Physiological Response to Ethanol of Rat Basolateral Amygdala Neurons Following Single-Prolonged Stress

**DOI:** 10.3389/fncel.2018.00219

**Published:** 2018-07-31

**Authors:** Laura C. Ornelas, N. B. Keele

**Affiliations:** Department of Psychology and Neuroscience, Baylor University, Waco, TX, United States

**Keywords:** basolateral amygdala, single-prolonged stress, ethanol, excitability, sex differences

## Abstract

Females are more likely to develop post-traumatic stress disorder (PTSD) than males. Also, symptoms of PTSD frequently precede alcohol abuse in females. Stressful, threat-related stimuli are evaluated by the amygdala, which is critical for establishing the emotional salience of environmental stimuli. Ethanol and stress have been shown to modify amygdala excitability, but effects of acute ethanol on neurons of the basolateral amygdala (BLA) in both males and females exposed to stress is unknown. The purpose of this study is to determine stress-induced changes in membrane properties of BLA neurons and to determine how ethanol modulates these changes in male and female rats. Whole-cell recordings were obtained from BLA neurons of both male and female rats exposed to single-prolonged stress (SPS). Neuronal excitability, quantified as the number of action potentials, was determined in current clamp mode by applying a series of depolarizing current steps. Hyperpolarization-activated current (*I*_h_) was elicited in voltage clamp. Excitability and *I*_h_ amplitude were determined before and during the superfusion of ethanol (EtOH; 30 mM) in BLA neurons from SPS-treated male and female rats. SPS alone did not alter the firing properties of BLA neurons from either males or females. However, following SPS, BLA neurons from males and females respond differently to ethanol. Superfusion of EtOH (30 mM) inhibited spike firing in BLA neurons from rats exposed to SPS, and EtOH-induced inhibition was greater in females than in males exposed to stress. Also, EtOH (30 mM) selectively decreased *I*_h_ amplitude in BLA neurons from SPS-treated male rats from 171 ± 46 pA in (pre-EtOH) control to 53 ± 51 pA in the presence of EtOH (30 mM). EtOH did not reduce *I*_h_ in BLA neurons from SPS-treated females. Together, these suggest important sex differences in the physiological responses to EtOH in stress disorders such as PTSD, that have high comorbidity with alcohol use disorders.

## Introduction

Post-traumatic stress disorder (PTSD) is a major public health concern. PTSD typically develops after exposure to potentially life-threatening events such as sexual assault, natural disasters, or combat exposure (DiMauro et al., 2014). Specific populations are at higher risk of developing PTSD based on greater likelihood to experience trauma. Females are more likely to develop PTSD compared to males in the general population (Kessler et al., 2005; Kilpatrick et al., 2013). In addition, development of PTSD more frequently precedes alcohol dependence in women than in men (Sonne et al., 2003). Also, women with PTSD and alcohol use disorders (AUDs) are more likely to consume alcohol to alleviate symptoms of stress and anxiety (Lehavot et al., 2014). Therefore, it is important to elucidate the cellular mechanisms that contribute to the roles of stress and anxiety that predispose to alcohol abuse.

The basolateral amygdala (BLA) plays a vital role in integrating sensory information and establishing emotional salience of the environment (Davis, 1992). The BLA receives direct excitatory transmission from long-range layer 2 pyramidal neurons in the prelimbic (PL) medial prefrontal cortex (mPFC) (Little and Carter, 2013). Dense with glutamatergic pyramidal neurons, the BLA projects reciprocal excitatory connections back to the mPFC, which are important in controlling stress and anxiety behaviors (Little and Carter, 2013). In addition, the BLA exerts direct activation of the central amygdala (CeA), the main output nucleus of the amygdala that drives neuroendocrine responses to stress and anxiety (Sah et al., 2003). This reciprocal interconnectivity significantly influences behavioral and endocrine responses to stress, anxiety and fear behavior. In addition, ethanol potentiates GABAergic inhibition of BLA neurons (Zhu and Lovinger, 2006), regulating glutamatergic excitatory transmission and playing an integral role in controlling anxiety-like behaviors (Silberman et al., 2008). GABAergic inhibition of the BLA occurs through two distinct pathways including GABAergic interneurons located in the BLA (Woodruff and Sah, 2007), and paracapsular intercalated cell masses including both lateral (lITC) and medial (mITC) clusters located in the external and internal capsule borders of the amygdala (Marowsky et al., 2005). While the effect of ethanol on the amygdala and fear behaviors is well-established, sex differences in the effects of ethanol on BLA neurophysiology and the effect of traumatic stress on ethanol-induced changes to BLA activity are currently unknown. Uncovering sex differences in BLA neuronal membrane properties in stressed animals may reveal potential neural mechanisms associated with resilience or susceptibility to stress, and improve individualized treatment.

To examine the effects of traumatic stress on BLA physiology of male and female rats, we utilized the single-prolonged stress (SPS) model of PTSD. Previously research has shown SPS model produces amplified acoustic startle response (Khan and Liberzon, 2004), longer time spent immobile during forced swim (Wu et al., 2016), and defensive reactions to and avoidance of trauma-related cues (Toledano and Gisquet-Verrier, 2016). In relation to neuroendocrine responses, SPS produces overactive and abnormal HPA-axis feedback (Liberzon et al., 1997). For example, SPS increases negative feedback of corticosterone (Kohda et al., 2007), increases glucocorticoid receptor expression (Liberzon et al., 1997), and produces a biphasic effect of mineralocorticoid receptor expression in the mPFC (Zhang et al., 2012). This constellation of SPS-induced changes is similar to human PTSD symptomology. Together the behavioral and neuroendocrine changes following SPS suggest physiological changes in the amygdala may be involved. However, the SPS model has yet to be used in electrophysiological studies examining BLA neuronal excitability in female rodents, or in males or females following ethanol exposure. Determining how ethanol affects excitability of BLA neurons of males and females after SPS will yield important new information about comorbid anxiety and alcohol use disorders and reveal important differences between males and females in their response to stress.

The purpose of this study is to determine stress-induced changes in membrane properties in BLA neurons and to determine how ethanol modulates these changes in male and female rats. We found that action potential firing in the BLA was similar in rats subjected to SPS and unstressed control rats, in both males and females. However, ethanol significantly decreased action potential firing in BLA neurons exposed to SPS in females only, suggesting that the inhibitory effect of alcohol is greater in females than in males exposed to stress. In contrast, ethanol reduced the amplitude of hyperpolarization-activated current (*I*_h_) only in BLA neurons from males exposed to SPS, in part because *I*_h_ amplitude is larger in BLA neurons from males than it is in females. These differences may contribute to the sex differences in comorbid PTSD and alcohol abuse.

## Materials and Methods

### Animals

All animal procedures were conducted according to a protocol approved by the Institutional Animal Care and Use Committee of Baylor University. Sprague-Dawley were bred in-house in the Baylor vivarium. Breeding was accomplished using triads of one male and two females, all approximately 6 months old at the time of breeding. One male was introduced to the female cage and remained until a vaginal plug was observed in both females, or for 1 week, whichever was shorter. On day postnatal day 21 (P21) the offspring were weaned, then group housed with same-sex littermates until sacrificed for the preparation of brain slices. All animals had *ad libitum* access to food and water and were maintained on a 12-h light/dark cycle. To control for litter effects, only one animal of either sex from each litter was used in either the control or SPS group. This resulted in a two-factor design where factor 1 is sex (male or female) and factor 2 is stress (control or SPS).

### Single-Prolonged Stress (SPS)

Both male and female rats underwent SPS. Animals were between the ages of P25 and P50 (approximately 60–120 g) when subjected to the SPS procedures. Animals in the SPS group were subjected to 2-h whole body restraint in an animal holder, followed immediately by 20 min of forced swim stress. Exactly 15 min after completion of the swim stress, animals lost consciousness by inhalation of ether (Liberzon et al., 1997; Ding et al., 2010; Ganon-Elazar and Akirav, 2012). Restraint was accomplished by immobilizing animals inside a cylindrical plastic tube (Med Associates, Inc.) that was 7.5 cm in diameter × 19 cm long. Restraint tubes were placed on a hard surface atop a clean, dry absorbent pad. Animals were confined individually and continuously for 2 h in the restraint tubes. Forced swim stress was conducted by placing animals in a plastic cylinder 50.5 cm tall × 20 cm in diameter that had been filled with tap water to a depth of 28 cm and left to reach room temperature of approximately 22°C. Animals were placed in the pool facing the wall of the cylinder and left to swim for 20 min. After forced swim, animals were gently removed and placed into a dry bath towel to dry the entire body surface of the animal then placed in a clean, dry cage. Loss of consciousness was accomplished by exposing animals to diethyl ether. Ether (3 mL) was pipetted onto a cotton ball and placed in a 50 mL centrifuge tube that was placed inside a plastic cylinder containing a slotted keeper to retain the animal within the tube. The animal was gently placed into the restraint cylinder and exposed to the ether-soaked cotton ball for 5 min. Animals were then removed from the cylinder and placed in their home cage to recover from loss of consciousness. Following SPS, animals were individually housed where they remained for 7 days to allow for neuropathological changes to occur in response to SPS (Yamamoto et al., 2009). Animals in the control group were age-matched littermates of the SPS animals. Unstressed control animals remained in their home cage and were undisturbed except for normal animal husbandry.

### Tissue and Slice Preparation

Brain slices were prepared for electrophysiological recording from SPS animals 8 days after the SPS procedure, and from unstressed control rats. The distribution of ages of animals used is shown in Supplementary Figure S1. Rat brains were rapidly removed and placed in cold (4°C) low calcium artificial cerebrospinal fluid (low-Ca^2+^ aCSF) containing (in mM): NaCl (104), KCl (4.7), MgCl_2_ (6), NaH_2_PO^4^ (1.2), CaCl_2_ (0.5), glucose (11.5), and NaHCO_3_ (25), aerated with 95% O_2_, 5% CO_2_ mix. Brains were blocked by two coronal cuts (one anterior to the cerebellum, one posterior to the optic chiasm), and one horizontal cut to remove the cortex superior to the hippocampus. A midsagittal cut separated the hemispheres. Slices (500 μm) containing the amygdala were made using a vibratome. Slices remained in low Ca^2+^ aCSF for at least 1 h to acclimate to room temperature before recording (Keele et al., 1997; Keele and Randall, 2003).

### Electrophysiological Recordings

Brain slices containing the BLA were transferred to a recording chamber where they were continuously superfused with control aCSF that contained (in mM), NaCl (117), KCl (4.7), MgCl_2_ (1.2), NaH_2_PO4 (1.2), CaCl_2_ (2.5), glucose (11.5), and NaHCO_3_ (25), aerated with 95% O2, 5% CO_2_. The temperature in the recording chamber was maintained at 30 ± 1°C.

Recording electrodes of 2–5 MΩ tip resistance were pulled from borosilicate glass capillary tubing (Drummond Scientific) using a Flaming-Brown puller (Sutter Instruments). The internal solution consisted of (in mM): potassium-gluconate (122), NaCL (5.0), MgCl_2_ (2.0), CaCl_2_ (0.3), EGTA (1.0), HEPES (10.0), Na_2_ATP (5.0), Na_3_GTP (0.4). The calculated liquid junction potential was +16.7 mV, which was corrected prior to experiments using pClamp software (Figi et al., 2003). The blind approach (Blanton et al., 1989) was used to conduct whole-cell recordings from BLA neurons. After obtaining a tight seal (resistance >1 GΩ) the neuronal membrane was ruptured and neurons were voltage-clamped at a holding potential of −60 mV. All data were collected using pClamp software (v9; Molecular Devices). Only neurons that exhibited a resting membrane potential (RMP) of at least −50 mV and action potentials that overshoot 0 mV were included for analysis.

Whole-cell recordings were performed in both current and voltage clamp. Current/voltage (*I*/*V*) relationships were obtained in voltage clamp mode from a holding potential (*V*_h_) of −60 mV by applying a series of depolarizing and hyperpolarizing steps between −40 and −120 mV (400 ms duration). The steady-state membrane resistance was determined from a fit of the linear region of a single *I*/*V* relationship in each treatment condition, evoked by voltage steps between −50 and −70 mV. Resting membrane potential (RMP) was also measured from fitting the linear region of the *I*/*V* relationship as the membrane potential where *I* = 0.

The hyperpolarization-activated, cyclic nucleotide-gate current (*H*-current, *I*_h_) was determined using voltage clamp recordings. *I*_h_ was elicited from a holding potential of −40 mV by applying a series of hyperpolarizing voltage steps between −40 and −110 mV (2 s duration). *I*_h_ is calculated from a single voltage protocol by subtracting instantaneous current from steady state current (*I*_ss_ − *I*_i_ = *I*_h_, see **Figure 2G**). Instantaneous current is measured immediately following the decay of the capacitive transient current; approximately 5–20 ms after the step-change in *V*_m_. Steady state is measured at the end of each trace. The voltage-dependence of *I*_h_ activation was determined by normalizing the amplitude of *I*_h_ evoked at each hyperpolarizing step (*I*_o_) to the maximum current elicited from hyperpolarization to −110 mV (*I*_max_). *I*_o_/*I*_max_ was plotted as a function of membrane potential, and the data were fit to the Boltzmann equation *Y* = *A*+ (*B* − *A*)/(1+*e*^[(^*^V^*^1/2-^*^V^*^m)/^*^k^*^]^), where *Y* is the normalized current *I*_o_/*I*_max_, *A* is the maximum conductance (constrained to *A* = 1); *B* is the minimum conductance (constrained to *B* = 0), *V*_1/2_ is the voltage at which the conductance is half-activated, and k is the steepness of the curve. Data were fit using a least squares non-linear regression with Prism (ver. 5, GraphPad Software, La Jolla, CA, United States). Goodness of fit was determined by calculating the total variance (*R*^2^) of *I*_o_/*I*_max_ explained by the fit (*R*^2^ = 1 − SS_fit_/SS_T_, where SS_fit_ is the sum of squares of the best-fit non-linear regression and SS_T_ is the total sum of squares).

Current clamp recordings were used to determine the active properties of BLA neurons. Action potentials were elicited by applying depolarizing current steps between −50 and 450 pA (600 ms duration). Excitability was quantified as the number of spikes elicited during each depolarizing current step. Action potential (AP) threshold, AP amplitude, AP half-width and latency to the first AP spike were determined in current clamp and obtained from the first action potential fired with the lowest stimulation current.

The time constant for membrane charging (τ_m_) was also determined in current clamp by an exponential fit of the initial 30 ms voltage response to injecting 50 pA hyperpolarizating current.

### Estrous Cycle Monitoring

Vaginal smears were obtained from all female rats to assess phase of estrous cycle immediately before collecting tissue for electrophysiological recordings. Estrous cycle synchronization across female rodents was unattainable. Therefore, to maximize animal availability, neurons from females of all phases of the estrous cycle were included in analyses.

### Ethanol Superfusion

EtOH (30 mM) was added to the aCSF bath solution and applied by superfusion. Passive and active membrane properties of BLA neurons are recorded prior to the superfusion of ethanol (control) and again following at least 10 min of ethanol superfusion.

### Statistical Analysis

To determine SPS-induced changes in neuronal excitability and *I*_h_ amplitude, data were analyzed by two-factor analysis of variance (ANOVA) with the following factors (levels): factor 1 is sex (male, female), factor 2 is SPS (Control, SPS). To determine ethanol-induced changes in neuronal excitability and *I*_h_ amplitude in SPS animals, we applied two-factor ANOVA with the following factors (levels): factor 1 is sex (male, female), factor 2 is ethanol treatment (Control, Ethanol) and treated as a repeated measure. In addition, we analyzed membrane properties and *I*_h_ amplitude by analysis of covariance (ANCOVA) using postnatal age as a covariate. For all dependent variables, the covariate *F* statistic was less than 1 (*F* < 1.0; *p* > 0.05). Since the ANOVA tests provided greater power (more degrees of freedom), we report these tests without age as a covariate. *Post hoc* comparisons were determined using Student’s *t*-test, corrected for multiple comparisons. The acceptable level of type I error was *p* < 0.05.

## Results

### Single-Prolonged Stress Does Not Alter Passive Membrane Properties of BLA Neurons From Either Male or Female Rats

Single-prolonged stress produced no changes in resting membrane potential (RMP), input resistance (*R*_in_), or the membrane time constant (τ_m_) (**Table 1**). Two-way ANOVA reveal no significant main effects or significant sex × SPS interactions on any of the resting membrane properties examined. For RMP, there was no significant main effect of sex [*F*(1,36) = 0.01, *p* > 0.05] or SPS [*F*(1,36) = 0.57, *p* > 0.05], and no sex × SPS interaction [*F*(1,36) = 1.13, *p* > 0.05]. For input resistance (*R*_in_), there was no significant main effect of sex [*F*(1,36) = 0.54, *p* > 0.05] or SPS [*F*(1,36) = 2.18, *p* > 0.05], and no sex × SPS interaction [*F*(1,36) = 0.08, *p* > 0.05]. For tau (τ), there was no main effect of sex [*F*(1,36) = 3.03, *p* > 0.05] or SPS [*F*(1,36) = 2.57, *p* > 0.05], and no sex × SPS interaction [*F*(1,36) = 0.11, *p* > 0.05]. These data suggest SPS does not change electronic properties of BLA neurons in either male or female rats.

**Table 1:** Single-prolonged stress (SPS) had no conclusive effect on resting properties of BLA neurons from male and female rats.

Sex	RMP (mV)		*R*_in_ (MΩ)		τ (ms^−1^)	
SPS	Male	Female	Male	Female	Male	Female
Control	−61 ± 3	−57 ± 2	172 ± 25	160 ± 14	21 ± 3	16 ± 2
SPS	−55 ± 4	−58 ± 4	143 ± 21	120 ± 26	16 ± 2	13 ± 2

*Data shown are membrane potential (RMP), input resistance (R_in_) and the membrane time constant (tau, τ) for male and female rats in both the Control and SPS conditions. ANOVA revealed no significant main effects or interaction for any of the variables examined. See text for details. Data are shown as mean ± SEM from 20 BLA neurons from males (Control, N = 13; SPS, N = 7) and 20 BLA neurons from females (Control, N = 12; SPS, N = 8).*

### Single-Prolonged Stress Alters Spike Latency but Not Action Potential Threshold, Amplitude or Half-Width of BLA Neurons From Either Male or Female Rats

Single-prolonged stress produced no changes in action potential threshold, action potential amplitude, or action potential half-width (**Table 2**). Two-way ANOVA revealed a significant main effect of stress in which SPS produced a delay in spike latency in male and female BLA neurons [*F*(1,26) = 5.31, *p* < 0.05]. There was no significant main effect of sex or sex × SPS interaction (*p* > 0.05). For action potential threshold, there was no significant main effect of sex [*F*(1,28) = 2.10, *p* > 0.05], or SPS [*F*(1,28) = 0.01, *p* > 0.05], and no sex × SPS interaction [*F*(1,28) = 1.60, *p* > 0.05]. For action potential amplitude, there was no significant main effect of sex [*F*(1,27) = 0.06, *p* > 005], SPS [*F*(1,27) = 0.43, *p* > 0.05], and no sex × SPS interaction [*F*(1,27) = 0.33, *p* > 0.05]. For action potential half-width, there was no significant main effect of sex [*F*(1,27) = 2.70, *p* > 0.05], SPS [*F*(1,27) = 0.57, *p* > 0.05], and no sex × SPS interaction [*F*(1,27) = 1.78, *p* > 0.05].

**Table 2:** Single-prolonged stress (SPS) has no conclusive effect on action potential properties of BLA neurons from male and female rats.

Sex	AP threshold (mV)		AP amplitude (mV)		AP half-width (ms)		Spike latency (ms)	
SPS	Male	Female	Male	Female	Male	Female	Male	Female
Control	36 ± 1	28 ± 3	75 ± 4	71 ± 3	1.46 ± 0.08	1.90 ± 0.15	21 ± 5	26 ± 8
SPS	32 ± 3	32 ± 5	75 ± 6	71 ± 9	1.54 ± 0.23	1.59 ± 0.17	45 ± 10	54 ± 24

*Data shown are action potential (AP) threshold, action potential amplitude, action potential half-width and spike latency for male and female rats in both the Control and SPS conditions. ANOVA revealed one main effect of stress on spike latency, in which SPS caused a delay in spike latency in both males and female (p < 0.05; see text for details). Data are shown as mean ± SEM.*

### Single-Prolonged Stress Does Not Alter Action Potential Firing of BLA Neurons From Either Male or Female Rats

**FIGURE 1:**
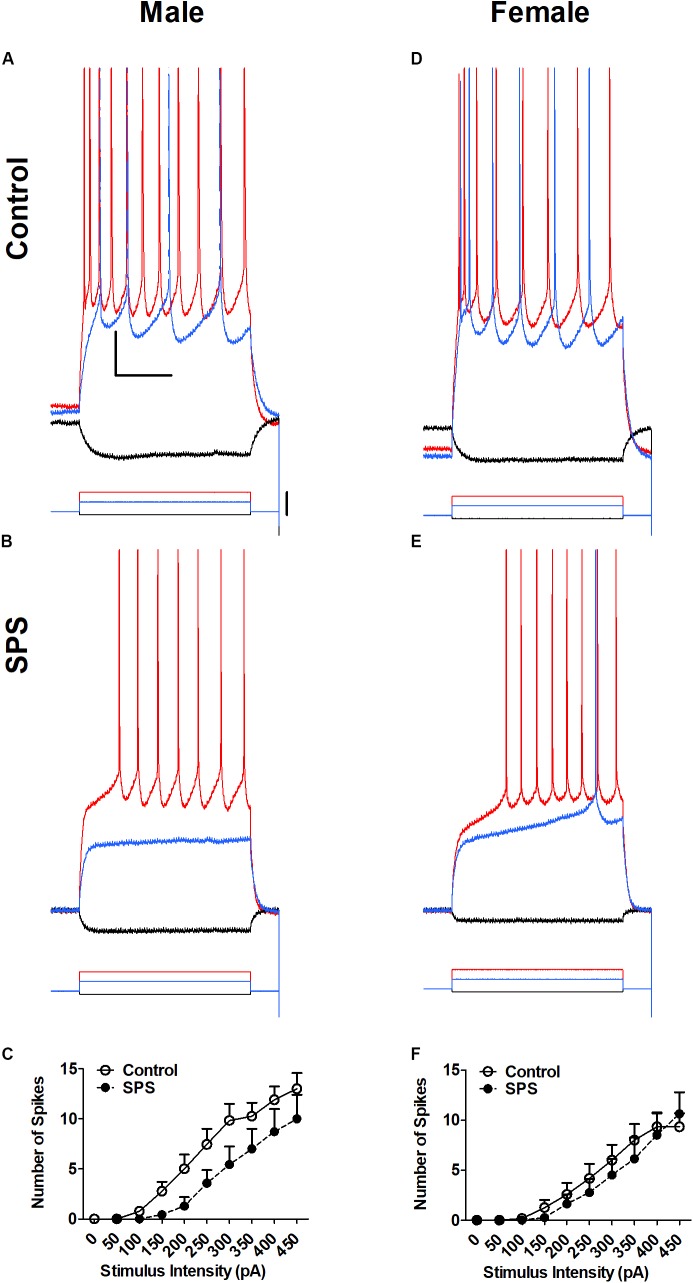
Single-prolonged stress (SPS) did not change neuronal excitability of basolateral amygdala (BLA) neurons from male **(A–C)** or female **(D–F)** rats. Representative action potential firing in BLA neurons from rats in the unstressed control (Control, **A,D**) and single prolonged stress (SPS, **B,E**) conditions. Action potentials were elicited form a holding potential of –60 mV by a series of depolarizing current steps. Responses to –50 pA hyperpolarizing current (black), 150 pA (blue) and 300 pA (red) depolarizing current are shown in **(A,B)** (Male). Responses to –50 pA hyperpolarizing current (black), 200 pA (blue), and 300 pA (red) depolarizing current are shown and **(D,E)** (Female). Peaks of the action potentials are truncated at +30 mV. Summary data from all neurons are show in **(C)** (Males: Control, *N* = 13; SPS, *N* = 7) and **(F)** (Females: Control, *N* = 12; SPS, *N* = 8). Data are show as mean ± SEM. Two-way ANOVA revealed no significant main effects of sex or SPS, and no sex × SPS interaction (see text for details).

Neuronal excitability of BLA neurons was determined in brain slices from both male and female rats (**Figure 1**). Excitability was measured in current clamp mode as the number of action potential spikes elicited by depolarizing current injection. Representative recordings obtained in control aCSF are shown in **Figure 1A** (male) and **Figure 1D** (female). Representative recordings obtained in rats exposed to SPS are shown in **Figure 1B** (male) and **Figure 1E** (female). Summary input-output relationships from all neurons are shown in **Figure 1C** (male) and **Figure 1F** (female). Two-way ANOVA revealed no significant main effect of sex [*F*(1,36) = 1.24, *p* > 0.05] or stress [*F*(1,36) = 2.48, *p* > 0.05] and no significant sex × stress interaction [*F*(1,36) = 1.03, *p* > 0.05].

### Sex Differences in Hyperpolarization-Activated Current (*I*_h_) in BLA Neurons From Males and Females

**FIGURE 2:**
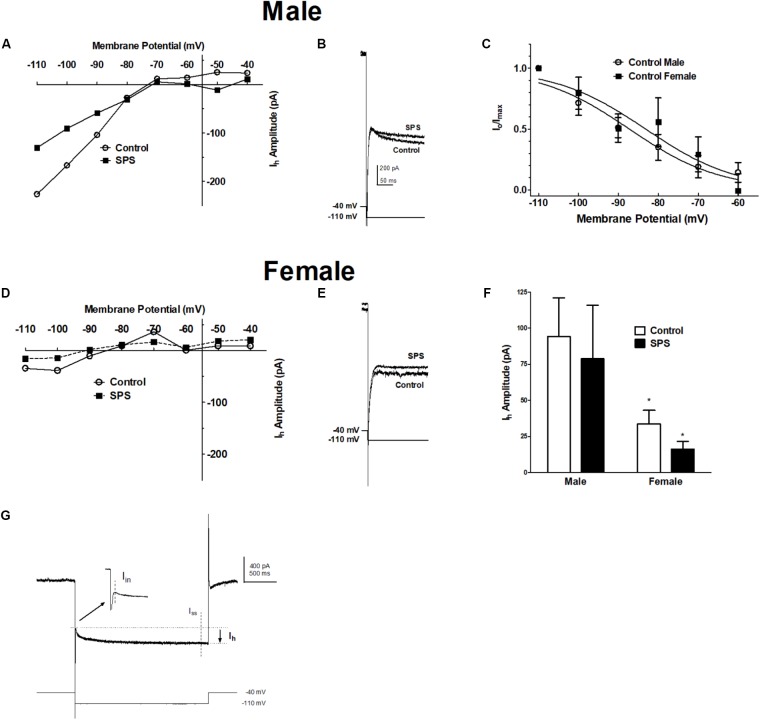
Single-prolonged stress does not alter the amplitude of the hyperpolarization-activated current (*I*_h_) in BLA neurons from either male or female rats. *I*_h_ was elicited from a holding potential of –40 mV by a series of hyperpolarizing voltage steps in 10 mV increments (2 s duration) to –110 mV. **(A)** Current-voltage relationships of *I*_h_ obtained from representative BLA neurons from male rats in the unstressed control (Control, 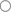) and single prolonged stress (SPS, 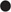) conditions. **(B)** Representative recordings of *I*_h_ elicited upon hyperpolarization to –110 mV in BLA neurons from male SPS or control male rats. **(C)** Activation curves of *I*_h_ in BLA neurons from Control male and female rats show no sex differences in voltage-dependence of the conductance underlying *I*_h_. Current-voltage relationships and representative *I*_h_ recordings in BLA neurons from female Control and SPS rats are show in **(D,E)**, respectively. Scale in **(B)** is the same in **(E)**. **(F)** The amplitude of *I*_h_ evoked upon hyperpolarization to –110 mV in males (left; Control, *N* = 13, open bars; SPS, *N* = 7, filled bars) and females (right; Control, *N* = 12; SPS *N* = 6). **(G)** Shows a representative current elicited upon 2 s hyperpolarization from –40 to –110 mV. *I*_h_ is calculated as the difference between the instantaneous current (*I*_in_) and the steady-state current (*I*_ss_). Data are shown as mean ± SEM ^∗^*p* < 0.05, compared to male/control condition (ANOVA followed by Student’s *t*-test. See text for details).

To examine further the active properties of BLA neurons in response to SPS, we determined the effect of SPS on the hyperpolarization-activated, cyclic nucleotide-gated cation current, *I*_h_ (**Figure 2**). *I*_h_ was elicited in voltage clamp mode by a series of hyperpolarizing steps (2 s duration) from a holding potential of −40 mV. Two-way ANOVA revealed a significant main effect of sex [*F*(1,34) = 6.03, *p* < 0.05] but no main effect of SPS [F(1,34) = 0.43, *p* > 0.05], and no significant sex × SPS interaction [*F*(1,34) = 0.01, *p* > 0.05]. The significant main effect of sex remained when we controlled for postnatal age [*F*(1,31) = 4.32, *p* < 0.05] (Supplementary Figure S1). *Post hoc* comparisons showed that *I*_h_ amplitude in BLA neurons from male rats was significantly larger than *I*_h_ in neurons from females (**Figure 2F**) [male: 94 ± 27 pA; female: 34 ± 9 pA; *t*(33) = 2.07, *p* < 0.05]. We also examined the voltage-dependence of activation of *I*_h_ in BLA neurons from both male and female rats in the unstressed control group (**Figure 2C**). The *I*_h_ current amplitude elicited at each voltage step (*I*_o_) was normalized to the maximal current (*I*_max_) elicited from hyperpolarization to −110 mV. *I*_o_/*I*_max_ was fit with the Boltzman equation (see section “Materials and Methods”). In BLA neurons from control rats, the goodness of fit of activation data were *r*^2^ = 0.5 for males and *r*^2^ = 0.3 for females. The activation curves show the conductance underlying *I*_h_ begins to open with hyperpolarization to approximately −70 mV. In BLA neurons from unstressed control rats, the half-maximal voltage of activation (*V*_1/2_) was −87 ± 2 mV in males, and −83 ± 4 mV in neurons from female rats [*t*(17) = 0.58, *P* > 0.05]. The decreased amplitude of *I*_h_ observed in females is not due to a difference in the voltage-dependence of activation. Together, these data confirm our previous findings that *I*_h_ is smaller in BLA neurons from female rats, and extend these finding by showing that SPS does not produce addition inhibition of *I*_h_.

### Ethanol (EtOH) Does Not Affect Passive Membrane Properties of BLA Neurons From SPS-Treated Rats

Ethanol-induced changes in resting membrane potential (RMP), input resistance (*R*_in_), and the membrane time constant (τ_m_) were determined in BLA neurons from both male and female SPS-treated rats (**Table 3**). Two-way ANOVA reveal no significant main effects or significant sex × SPS interactions on any of the resting membrane properties examined. For RMP, there was no significant main effect of sex [*F*(1,12) = 0.25, *p* > 0.05] or EtOH [*F*(1,12) = 3.0, *p* > 0.05], and no significant sex × EtOH interaction [*F*(1,12) = 0.42, *p* > 0.05]. For input resistance (*R*_in_), there was no significant main effect of sex [*F*(1,12) = 1.05, *p* > 0.05] or EtOH [*F*(1,12) = 0.04, *p* > 0.05], and no significant sex × EtOH interaction [*F*(1,12) = 0.23, *p* > 0.23]. For the membrane time constant Tau (τ), there was no significant main effect of sex [*F*(1,12) = 1.46, *p* > 0.05] or EtOH [*F*(1,12) = 1.8, *p* > 0.05], and no significant sex × EtOH interaction [*F*(1,12) = 0.89, *p* > 0.05]. These data suggest that EtOH does not change electronic properties of BLA neurons from either male or female SPS-treated rats.

**Table 3:** Ethanol (EtOH, 30 mM) had no conclusive effect on membrane properties of BLA neurons from SPS-treated male or female rats.

Sex	RMP (mV)		*R*_in_ (MΩ)		τ (ms^−1^)	
EtOH	Male	Female	Male	Female	Male	Female
Control	−64 ± 4	−63 ± 2	117 ± 9	135 ± 28	21 ± 3	14 ± 3
EtOH	−52 ± 8	−58 ± 5	108 ± 8	156 ± 39	14 ± 2	13 ± 3

*Data shown are membrane potential (RMP), input resistance (R_in_), and the membrane time constant (tau, τ) for Male and Female rats, both before (Control) and during superfusion of ethanol (EtOH, 30 mM). ANOVA revealed no significant main effects or interaction for any of the variables examined. See text for details. Data are shown as mean ± SEM from N = 3 BLA neurons from SPS-treated male rats, and N = 5 BLA neurons from SPS-treated female rats.*

### Ethanol Does Not Alter Active Membrane Properties of BLA Neurons From SPS-Treated Rats

Ethanol produced no changes in action potential threshold, action potential amplitude, action potential half-width, or spike latency (**Table 4**). However, two-way ANOVA revealed a significant main effect of sex on action potential half-width, in which females exposed to SPS produced significantly longer half-widths compared to males [*F*(1,12) = 4.92, *p* < 0.05]. There was no significant main effect of ethanol or ethanol × sex interaction (*p* > 0.05). For action potential threshold, amplitude, or spike latency, there was no main effect of sex, ethanol, or sex × ethanol interaction (*p* > 0.05).

**Table 4:** Ethanol (EtOH, 30 mM) had no conclusive effect on active membrane properties of BLA neurons from SPS-treated male or female rat.

Sex	AP threshold (mV)		AP amplitude (mV)		AP half-width (ms)		Spike latency (ms)	
EtOH	Male	Female	Male	Female	Male	Female	Male	Female
Control	35 ± 1	27 ± 5	84 ± 6	73 ± 6	1.32 ± 0	1.68 ± 0.20	50 ± 15	98 ± 65
EtOH	29 ± 3	27 ± 4	67 ± 9	65 ± 4	1.48 ± 0.18	2.11 ± 0.26	40 ± 21	72 ± 28

*Data shown are action potential (AP) threshold, action potential amplitude, action potential half-width and spike latency for male and female rats in both the Control and SPS conditions. ANOVA revealed one main effect of sex on action potential half-width, in which females have longer action potential half-widths compared to males (p < 0.05; see text for details). Data are shown as mean ± SEM.*

### Ethanol Decreases Neuronal Excitability in BLA Neurons From SPS-Treated Rats Differently in Males and Females

**FIGURE 3:**
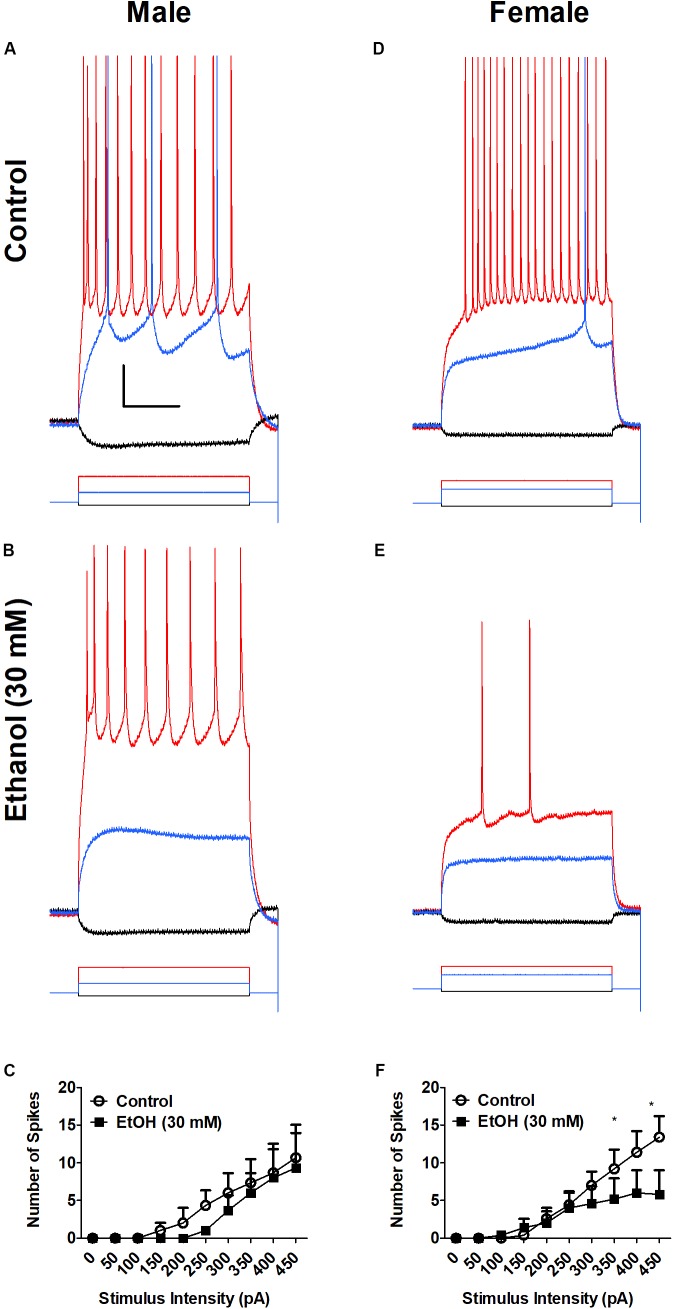
Ethanol (EtOH) decreases neuronal excitability of BLA neurons from SPS female **(D–F)**, but not male **(A–C)** rats. Representative action potential firing in BLA neurons from SPS-treated rats before (Control, **A,D**) and during superfusion of EtOH (30 mM, **B,E**). Action potentials were elicited from a holding potential of –60 mV by a series of depolarizing current steps. Responses to –50 pA hyperpolarizing current (black), 150 pA (blue) and 400 pA (red) depolarizing current are shown in **(A,B)** (Male). Responses to –50 pA hyperpolarizing current (black), 200 pA (blue) and 400 pA (red) depolarizing current are shown and **(D,E)** (Female). Peaks of the action potentials are truncated at +30 mV. Summary data from all neurons are shown in **(C)** (Males, *N* = 3) and **(F)** (Females, *N* = 5). Data are shown as mean ± SEM. Two-way ANOVA showed a significant main effect of EtOH, but no main effect of sex and no sex × EtOH interaction (see text for details). ^∗^*p* < 0.05, EtOH compared to control at the same stimulus intensity (ANOVA followed by Student’s *t*-test. See text for details).

While the effect of ethanol on the amygdala and fear behaviors is well-established, sex differences in the effects of ethanol on BLA neurophysiology after traumatic stress are currently unknown. Therefore, we examined the effects of acute ethanol on BLA excitability in SPS-treated rats (**Figure 3**). Males were PD 42 ± 5 and females were PD 41 ± 6 on the day of recording. Ethanol (30 mM) decreases action potential firing in SPS male (**Figures 3A,B**) and female (**Figures 3D,E**) rats following depolarizing current steps (200–450 pA, 600 ms). There was no significant main effect of sex (*p* > 0.05). However, there was a significant main effect of ethanol [*F*(1,66) = 12.51, *p* < 0.01]. With strong depolarization (400–450 pA), superfusion of ethanol (30 mM) significantly decreased spike firing in BLA neurons from females only [400 pA step *t*(6) = 2.4, *p* > 0.05; 450 pA step *t*(6) = 3.4, *p* > 0.01]. EtOH also decreased firing at smaller depolarizing steps in BLA neurons from males but this was not significant (*p* > 0.05). There was no sex × EtOH interaction [*F*(1,66) = 1.19, *p* > 0.05]. These data show that EtOH-induced inhibition is greater in BLA neurons from SPS-treated female than in SPS-treated male rats.

### Ethanol Inhibits Hyperpolarization-Activated Current (*I*_h_) in BLA Neurons From SPS-Treated Rats

**FIGURE 4:**
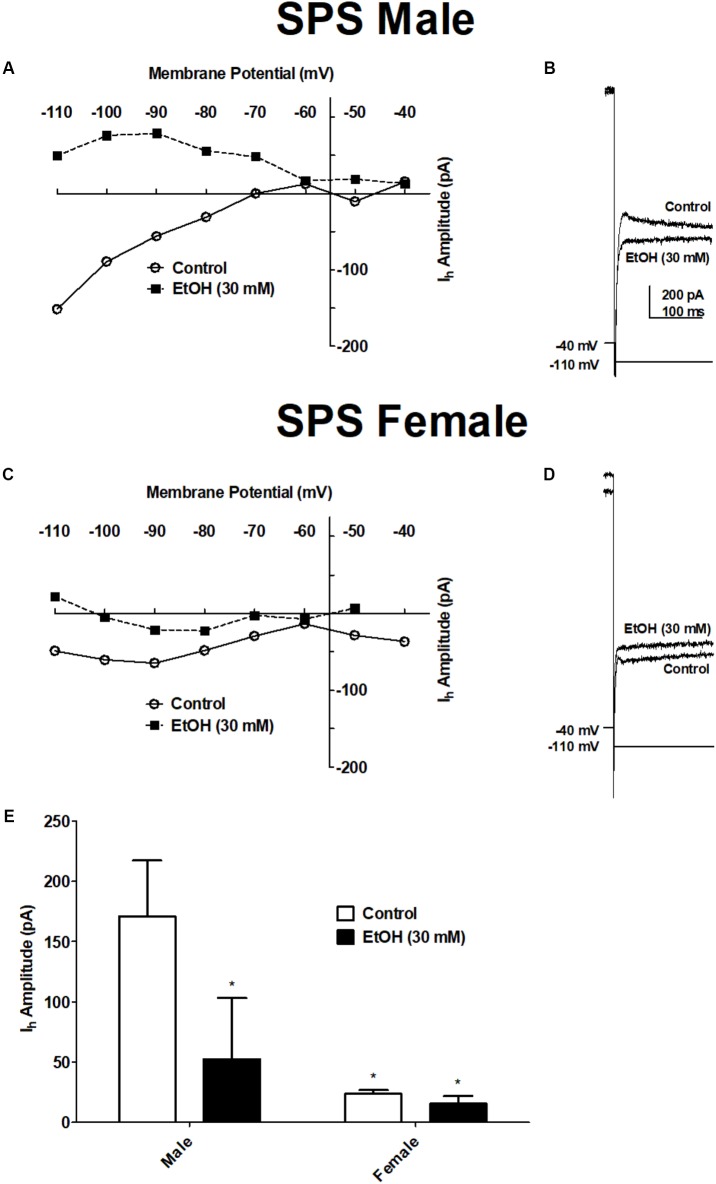
Ethanol decreased the amplitude of the hyperpolarization-activated current (*I*_h_) in BLA neurons from SPS-treated male but not SPS-treated female rats. *I*_h_ was elicited from a holding potential of –40 mV by a series of hyperpolarizing voltage steps in 10 mV increments (2 s duration) to –110 mV. **(A)** Current–voltage relationships of *I*_h_ obtained before (Control, 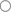) and during ethanol (EtOH 30 mM; 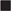) in a typical BLA neuron from a male SPS-treated rat. **(B)** Representative recordings of *I*_h_ elicited upon hyperpolarization to –110 mV before (Control) and during EtOH (30 mM) from the same neuron shown in **(A)**. Current-voltage relationships and representative *I*_h_ recordings in BLA neurons from a female SPS-treated rat are shown in **(C,D)** respectively. Scale in **(B)** is the same in **(D)**. **(E)** The amplitude of *I*_h_ evoked upon hyperpolarization to –110 mV in males (left, *N* = 3) and females (right, *N* = 4) before (Control, open bars) and during superfusion of EtOH (30 mV, filled bars). Data are shown as mean ± SEM. ^∗^*p* < 0.05 compared to male Control condition (ANOVA followed by Student’s *t*-test. See text for details).

Previously we showed that EtOH reduces hyperpolarization-activated, cyclic nucleotide-gated cation current (*I*_h_) in the BLA (Ornelas et al., 2017). In the present study, we examined EtOH-induced inhibition of *I*_h_ in BLA neurons from SPS-treated rats to determine sex differences in BLA excitability following acute ethanol. *I*_h_ was elicited in voltage clamp mode by a series of hyperpolarizing steps (2 s duration) from a holding potential of −40 mV. *I*_h_ was elicited in BLA neurons from both SPS-treated male (PD 42 ± 5 days) and female (PD 41 ± 6 days) rats, before (Control) and during superfusion of EtOH (30 mM). Results are show in **Figure 4**. Ethanol decreases *I*_h_ amplitude in BLA neurons from both SPS-treated male and female rats. Two-way ANOVA revealed a significant main effect of EtOH [*F*(1,6) = 278.8, *p* < 0.0001] and a significant sex × EtOH interaction [*F*(1,6) = 211.9, *p* < 0.0001]. There was a marginal effect of sex (*p* = 0.07). *Post hoc* comparison showed that *I*_h_ amplitude in BLA neurons from female rats was significantly smaller than *I*_h_ in neurons from males [*t*(5) = 3.58, *p* < 0.05]. In BLA neurons from SPS-treated male rats, EtOH (30 mM) significantly decreased *I*_h_ amplitude from 171 ± 46 pA in control to 53 ± 51 pA (*n* = 3) in the presence of EtOH [*t*(1,5) = 20.67, *p* < 0.001]. Ethanol similarly decreased *I*_h_ amplitude in BLA neurons from female rats from 24 ± 3 pA to 16 ± 6 pA (*n* = 4 out of 5), but this was not significant (*p* > 0.05). Together, these data show that following SPS, EtOH inhibits *I*_h_ selectively in BLA neurons from SPS-treated male rats.

## Discussion

In the current study we sought to determine sex differe membrane excitability in response to SPS and acute ethanol. SPS does not alter action potential firing in BLA pyramidal neurons from either male or female rats. In addition, SPS does not produces changes to hyperpolarization-activated current (*I*_h_) in either males or females; however, BLA neurons in males overall have larger *I*_h_ amplitudes compared to females, suggesting *I*_h_ plays a reduced role in modifying neuronal activity in the amygdala in females. In response to acute ethanol, females exposed to SPS exhibited greater ethanol-induced inhibition on action potential firing compared to SPS-treated males. However, *I*_h_ was reduced by ethanol only in SPS-treated males and not females exposed to stress. These results suggest SPS alone does not affect action potential firing or *I*_h_ in both males and females, but ethanol-induced inhibition of BLA excitability is greater in SPS-treated females than in SPS-treated males.

### SPS and Excitability of the BLA of Males and Females

The SPS model produces overactive and abnormal HPA axis feedback (Liberzon et al., 1997), amplified acoustic startle response (Khan and Liberzon, 2004), and fear responses to trauma-related stimuli (Wu et al., 2016). We therefore expected to find increased excitability in BLA neurons from SPS-treated rats. The BLA has been implicated in regulating anxiety-like behavior through direct excitatory projections to the central nucleus of the amygdala, in conjunction with top-down control provided by the mPFC. Recently, SPS produced suppression of long-term potentiation (LTP) in external capsule-lateral amygdala (EC-LA) synapses potentially producing disinhibition in pyramidal neurons in the LA and increased fear conditioning (Kohda et al., 2007). Although overactive amygdala activity has been shown in people with PTSD (Badura-Brack et al., 2018) and in animals exposed to stress (Hetzel and Rosenkranz, 2014), previous research has also shown SPS produces neuronal apoptosis in the amygdala (Liu et al., 2010) that may underlie decreased amygdala volume in individuals with PTSD (Karl et al., 2006). Furthermore, increased apoptosis could result in reduced action potential firing and decreased excitability of the BLA. SPS-induced changes in BLA excitability have been largely unexplored. Therefore, the data shown here represents an early investigation of SPS-induced changes in membrane properties of the BLA in both males and females.

Single-prolonged stress did not affect *I*_h_ amplitudes in BLA neurons from either males or females, but we did replicate previous findings that *I*_h_ is smaller in BLA neurons from females. Although *I*_h_ has been shown to play a role in membrane excitability in males (Ornelas et al., 2017), the small amplitude of *I*_h_ shown in BLA neurons from females suggests *I*_h_ is not a primary mechanism involved in controlling activity of the amygdala in females. In addition, we did not observe sex differences in BLA excitability in response to SPS, suggesting that SPS-induced changes in BLA excitability are not likely to contribute extensively to sex differences in other SPS-induced changes. Although we did not see sex differences in *I*_h_ in response to SPS, this study was one of the few to evaluate sex as a factor influencing susceptibility to SPS. Saffari et al. (2015) examined hippocampal BDNF protein levels in response to SPS and β-estradiol in both male and female rats. Results indicate the SPS model reduced hippocampal cell density and this reduction was partly corrected by β-estradiol; however, there were no sex-differences in conditioned-fear responses to SPS or treatment of β-estradiol. A second study that examined stress-induced effects of SPS between males and females was Keller et al. (2015), which was of the first study to show female rats are more resilient to the SPS model. For example, SPS-treated female rats exhibit less glucocorticoid expression in the ventral hippocampus and greater retention of cued fear extinction compared to males (Keller et al., 2015). It has been well-established that females are more likely to develop PTSD compared to males in the general population (Kessler et al., 2005; Kilpatrick et al., 2013). Therefore, the results from the current study and previous studies (Keller et al., 2015; Saffari et al., 2015) examining sex differences in response to the SPS model, indicate the importance and need for further research to establish generalizability of the SPS model across sexes.

### SPS and Ethanol-Induced Changes to BLA Neurons of Males and Females

The current study also examined neurophysiological effects of acute ethanol on BLA neurons in both male and females exposed to SPS. While the effect of ethanol on the amygdala and stress/anxiety is well established (Silberman et al., 2009; Gilpin et al., 2015; Morales et al., 2018), sex differences in the effects of ethanol on BLA neurophysiology and the effect of traumatic stress on ethanol-induced changes to BLA activity are currently unknown. Interestingly, ethanol-induced inhibition of action potential firing was greater in females exposed to SPS than in males. We have previously investigated the acute effects of ethanol in unstressed (control) rats (Keele et al., 2017). Previous results showed that ethanol-mediated inhibition is greater in males than in females. In both the medial (CeM) and lateral (CeL) nuclei, ethanol more strongly reduces excitatory postsynaptic potentials (EPSPs) in males than in females (Logrip et al., 2017), suggesting sex differences in sensitivity and responsivity to acute ethanol. In the current study we extend those findings here by determining the effect of acute ethanol in SPS-treated rats. Here we showed that ethanol has the opposite effect on excitability in SPS-treated rats, namely that ethanol-induced inhibition of BLA excitability is greater in females than in males.

The effects of ethanol reported here are consistent with a direct effect of ethanol on HCN channels underlying *I*_h_. Ethanol has well-known effects on both GABAA and NMDA receptors. For example, spontaneous GABAergic IPSCs in the BLA are increased by the presence of ethanol (Zhu and Lovinger, 2006), suggesting ethanol may be acting via GABA receptors to contribute to the inhibitory effects reported in the present study. Similarly, *I*_h_ in the BLA is modulated by the neuropeptides corticotropin-releasing factor (CRF) and neuropeptide Y (Giesbrecht et al., 2010), where NPY inhibits but CRF enhances *I*_h_. NPY-mediated inhibition of *I*_h_ is similar to our findings and raises the possibility that ethanol-induced inhibition of *I*_h_ may involve NPY Y1 receptor mechanisms. However, we did not observe changes in either membrane potential or membrane resistance during or after superfusion of ethanol. Since superfusion of ethanol did not change either membrane potential or membrane resistance, the ethanol-induced inhibition of *I*_h_ is not likely to involve postsynaptic conductance changes involving amino acid or peptide receptors. However, the specific mechanism involved in ethanol-mediated inhibition of *I*_h_ remains to be determined directly.

It is important to note that animals included in the current study fall within the rodent adolescent period. However, there were no differences in the effects of sex or SPS due to differences in the covariate, postnatal age. While neurodevelopmental changes occur during the rodent adolescent period (PD 28–50), the developmental regulation of HCN channel expression and *I*_h_ amplitude is stabilized during the early postnatal period in many brain areas. In the hippocampus, HCN channels develop through postnatal day (PD) 20 (Vasilyev and Barish, 2002). HCN channel expression and *I*_h_ current in PFC projection neurons from adolescents (PD 35 – 40) are similar in adults (PD 60 – 70) (Yang et al., 2018). However, these studies determined the developmental regulation of HCN channels in males, and few if any studies have investigated the development of HCN channels or *I*_h_ in females. Furthermore, physiological properties of BLA neurons on PD28 is very similar to neurons recorded after PD35 and maximal firing rates in BLA principal neurons reach maturity at PD14 (Ehrlich et al., 2012). Therefore, using ages of animals throughout the adolescent period should not result in differences between animals in set developmental stages. Altogether these data suggest that SPS and ethanol may be interacting directly with adult-like HCN channels to inhibit *I*_h_ amplitude.

### Acute Ethanol Inhibits *I*_h_ Differently in SPS-Treated Males and Females

We also showed that *I*_h_ is inhibited by ethanol more strongly in BLA neurons from SPS-treated males than SPS-treated females. We expected to find that either SPS and/or ethanol would have congruent effects on excitability and *I*_h_, namely that inhibition of excitability would also occur with lower amplitudes of *I*_h_. However, our data show that *I*_h_ is reduced by ethanol in SPS-treated males, but that BLA excitability is unchanged by ethanol; and conversely that excitability is inhibited by ethanol in SPS-treated females but *I*_h_ amplitude is not reduced by ethanol in SPS-treated females. This discrepancy was unexpected but may reveal novel sex-dependent effects of *I*_h_ on the regulation of neuronal excitability in the BLA. Previous studies of *I*_h_ in females have examined the role of *I*_h_ on cardiovascular and hormonal functioning (Arroyo et al., 2006; Han et al., 2014; He et al., 2015). However, within the amygdala, limited research has compared *I*_h_ between males and females. Understanding the differing role of *I*_h_ in BLA neurons improves our understanding of the sex-related differences cellular mechanisms underlying membrane excitability and ensuing amygdala-dependent behaviors. These data are consistent with a growing body of data showing that females with PTSD and alcohol use disorders are more likely to consume alcohol to alleviate symptoms of stress and anxiety (Lehavot et al., 2014). We suggest that the anxiolytic properties of ethanol may involve the inhibition of excitability and *I*_h_ in BLA neurons in male rats, but ethanol may be less anxiolytic in females because the inhibitory effect of ethanol on *I*_h_ is occluded by the low amplitude *I*_h_ observed in BLA neurons of females. This may contribute to increased ethanol consumption in females. These data are consistent with other findings that, in adolescent female rats, preference for alcohol and alcohol intake are increased after exposure to restraint stress (Wille-Bille et al., 2017). Furthermore, adolescent female rats with greater endogenous anxiety engage in more ethanol drinking and have stronger preference for alcohol (Acevedo et al., 2016) indicating age-related factors in alcohol-stress-anxiety interactions. Altogether, these data suggest that there are neurobiological mechanisms of vulnerability to ethanol during stressful events that are different in males and females, and that sex differences in ethanol-induced inhibition of *I*_h_ may contribute to sex differences in vulnerability to alcohol abuse following traumatic stress.

## Conclusion

The current study examined sex differences in the effects of ethanol on BLA neurophysiology and the effect of traumatic stress on ethanol-induced changes to BLA activity. Determining the physiological properties of amygdala neurons in the SPS model has not been widely accomplished in either male or female rats. This is therefore one of the first determinations of the effects of SPS neuronal excitability in the BLA. In response to ethanol, we have shown distinct sex differences in BLA membrane excitability in SPS-treated male and female rats. Ethanol reduced action potential firing in the BLA more in females compared to males, suggesting greater ethanol-induced inhibition in females exposed to traumatic stress. Furthermore, this study is one of the first to examine *I*_h_ in the BLA in males and females exposed to SPS and ethanol. Similar to our previous work, males exhibit significantly larger *I*_h_ amplitudes compared to females in response to stress and ethanol. Small *I*_h_ amplitudes in females may be associated with less neuronal excitability in response to ethanol. The sex differences controlling neuronal excitability in the BLA could perhaps contribute to sex differences in ethanol consumption following traumatic stress. Overall our data makes a significant impact to merging the current gap between sexes in neurophysiological studies by revealing important differences between sexes in cellular mechanisms that may contribute to alcohol abuse in stress and anxiety disorders.

## Author Contributions

LO and NK formulated the hypotheses, designed the experiments, analyzed the results, and revised the manuscript. LO conducted the experiments and wrote the original draft of the manuscript.

## Conflict of Interest Statement

The authors declare that the research was conducted in the absence of any commercial or financial relationships that could be construed as a potential conflict of interest.
