# Supplementary material for: Sex Differences in the Physiological Response to Ethanol of Rat Basolateral Amygdala Neurons Following Single-Prolonged Stress
Source: Front Cell Neurosci. 2018 Jul 31;12:219. doi: 10.3389/fncel.2018.00219 (PMC6079253; doi:10.3389/fncel.2018.00219)
Supplement: Supplementary file 1 [file Data_Sheet_1.PDF]

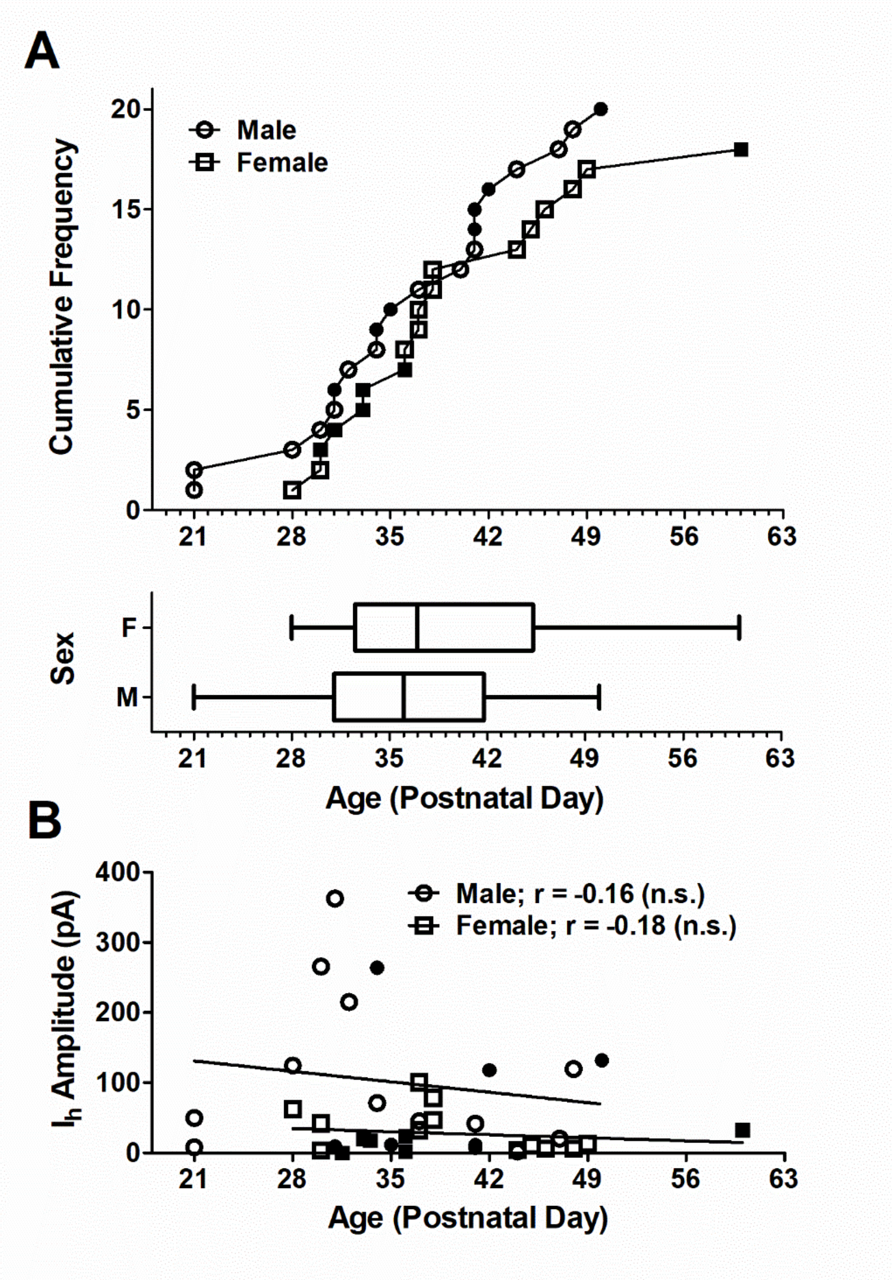

Figure S1

Figure S1. **A** illustrates the age of animals (PD) used to determine the effect of SPS on excitability of the basolateral amygdala (BLA) and the amplitude of hyperpolarization-activated current (I_h) in BLA neurons from males and females. The cumulative frequency histogram of animals used is shown in the upper figure. The lower figure shows a box and whisker plot, indicating the range, 1st and 3rd quartiles, and median age of males and females used. **B** shows the relationship between I_h amplitude postnatal age in both males and females. The data for males and females were fit separately by linear regression. The Pearson correlation coefficients are not significant. In both A and B, circles indicate males and squares indicate females. Open symbols indicated unstressed (Control) animals and filled symbols indicate animals subjected to single-prolonged stress (SPS).
